# Mechanical Property Evolution and Load Monitoring Method of Laminated Elastomeric Bridge Bearings Under Temperature Effects

**DOI:** 10.3390/s26103046

**Published:** 2026-05-12

**Authors:** Menglong He, Xianhui Liu, Nianchun Deng

**Affiliations:** 1 School of Civil and Architectural, Guangxi University of Science and Technology, Liuzhou 545006, China; hml010720@163.com (M.H.);; 2 School of Civil Engineering and Architecture, Guangxi University, Nanning 530004, China

**Keywords:** laminated elastomeric bearing, temperature effect, evolution of mechanical properties, laser displacement measurement system, load inversion

## Abstract

The mechanical behavior of laminated elastomeric bearings in service is highly sensitive to ambient temperature, whereas conventional monitoring approaches often fail to accurately capture their temperature-dependent load response. To address this issue, this study proposes a multi-temperature framework for identification and load monitoring of bridge elastomeric bearings. Using a high-precision laser displacement measurement system, six temperature levels were defined from −20 to 30 °C at 10 °C intervals. Room-temperature load–displacement calibration tests, compressive elastic modulus tests under different temperature conditions, and monitoring accuracy validation tests were then systematically conducted. Based on these experiments, the effects of temperature on the mechanical properties and compressive deformation response of the bearing were quantified, and an inverse load-identification model was developed. The results show that the compressive elastic modulus increases markedly with decreasing temperature, reaching a 32.11% increase at −20 °C relative to that at 30 °C. Under the same applied load, the vertical compressive deformation decreases significantly as temperature decreases, with a 27.76% reduction at −20 °C compared with that at 30 °C, indicating a pronounced low-temperature stiffening effect. The proposed inverse load-identification model achieves a maximum relative error of 4.83% over the full temperature range, demonstrating good accuracy and applicability. The proposed methodology provides a practical basis for mechanical-performance evaluation and high-precision monitoring of bridge bearings under complex thermal environments.

## 1. Introduction

Structural health monitoring (SHM) is a fundamental approach for ensuring the in-service safety and operational performance of bridges, and its effectiveness depends critically on the accurate perception of the working condition of key structural components [1,2,3]. As a vital force-transfer component connecting the superstructure and substructure, rubber bridge bearings play an essential role in transmitting vertical loads, accommodating girder rotation and horizontal displacement, and enhancing the seismic performance of the overall structure [4]. Owing to their favorable load-carrying capacity, deformation adaptability, and vibration/isolation performance, elastomeric bearings have been widely used in bridge and underground engineering applications [5,6,7,8]. However, because of the viscoelastic nature of rubber materials, the mechanical properties of bearings—including stiffness, damping, and deformation capacity—are highly sensitive to ambient temperature and exhibit pronounced temperature-dependent evolution during long-term service [9]. This temperature sensitivity not only alters the load characteristics of the bearing itself but also interferes with the accurate interpretation of monitoring data and the reliability of condition identification [10,11]. Therefore, investigating the temperature-dependent evolution of the mechanical behavior of bridge rubber bearings, together with corresponding load-monitoring methodologies, is of considerable importance for improving bearing condition assessment and bridge safety monitoring.

In addition to structural monitoring studies, recent research on intelligent fault diagnosis has increasingly emphasized robustness and generalization under limited data, unseen working conditions, and out-of-distribution scenarios. For example, Chen et al. [12] proposed a shrinkage Mamba relation network with out-of-distribution data augmentation for rotating machinery fault detection and localization under zero-faulty data conditions. Other studies have investigated category-invariant disentangled feature learning for domain generalization in machine fault diagnosis [13]. Although these studies focus on rotating machinery rather than bridge bearings, they highlight the importance of robustness, transferability, and generalization in monitoring models under varying operating conditions. These ideas are consistent with the motivation of the present study, in which temperature variation is considered as a key environmental factor affecting the reliability of bearing load monitoring.

At present, the performance evaluation and condition identification of bridge rubber bearings still face substantial challenges in terms of monitoring accuracy, long-term reliability, and practical applicability in engineering environments. Previous studies have shown that rubber materials are subject to aging during service, leading to continuous evolution in their mechanical properties and operational state over time, which imposes more stringent requirements on the long-term performance assessment of bearings [14]. To monitor the in-service behavior of bearings under coupled compression–shear loading, researchers have explored deep-learning-based approaches for identifying axial compression and shear deformation in rubber bearings, with promising progress also achieved in damage detection [15]. In parallel, some studies have incorporated embedded sensors or microsensing units into pot bearings, elastomeric bearings, and force-measuring bearings to enable real-time sensing of internal stress, strain, and load-related information [16]. Although these approaches can more directly capture local mechanical responses within the bearing, they still suffer from several limitations under complex service conditions, including insufficient sensor durability, restricted long-term stability, and difficulties in engineering implementation [17,18]. In addition, analytical investigations have shown that rubber bearings subjected to axial loading exhibit complex internal stress distributions and pronounced nonuniform deformation, such that a single monitoring index is often insufficient to comprehensively characterize their true load state and the evolution of early-stage damage [19,20]. Therefore, the development of more stable, accurate, and field-deployable methods for load monitoring and condition identification of bridge rubber bearings remains of significant engineering importance.

In studies of service-performance degradation and failure mechanisms of rubber bearings, the evolution of shear deformation and the identification of internal damage are central to condition assessment. Existing research has employed nondestructive monitoring techniques, such as active sensing, to detect internal rupture and shear deformation in rubber bearings, and to assess bearing safety by analyzing signal characteristics under different loading states, thereby providing an important foundation for intelligent bearing monitoring [21,22]. At the same time, the in-service behavior of bearings is jointly influenced by lateral stability, shear failure limits, and the coupling of complex loading effects; accordingly, their performance evaluation cannot rely solely on a single response indicator [23]. Further studies have shown that ambient temperature—particularly low temperature—can significantly alter the mechanical response of full-scale rubber bearings, amplifying their stiffness, yielding characteristics, and deformation behavior [24,25,26]. These macroscopic changes are fundamentally associated with underlying material mechanisms, such as low-temperature crystallization in rubber [27]. At the structural level, low temperature can also substantially affect the seismic response of lead-rubber bearings and isolated bridges, making parameter correction and temperature-adaptive design necessary in engineering practice [28]. In addition, natural rubber and synthetic rubber exhibit distinct thermomechanical coupling characteristics and service adaptability under low-temperature conditions, and related materials research has provided useful guidance for material selection and performance optimization of bridge rubber bearings in different environmental scenarios [29,30]. Therefore, although meaningful progress has been made in damage identification, shear-deformation monitoring, and low-temperature characterization of rubber bearings, systematic investigation into the coupling between temperature-dependent evolution and load-monitoring methods for bridge rubber bearings remains insufficient.

Ambient temperature can significantly affect the mechanical properties of laminated elastomeric bearings and their deformation response under sustained loading, thereby compromising the accuracy of bearing condition identification and structural health monitoring. To address the current lack of systematic research on the coupling between temperature-dependent evolution and load-monitoring methodologies for laminated elastomeric bearings, this study focuses on laminated elastomeric bearings and employs a high-precision laser displacement sensor to monitor their vertical compressive deformation under different temperature conditions. The measurement accuracy of the sensing system is validated against reference displacement-gauge data. Through compressive elastic modulus tests and sensor temperature tests conducted over multiple temperature levels, the influence of temperature variation on the mechanical behavior and deformation response of the bearing is comprehensively characterized. On this basis, a temperature-informed load inversion model is developed to enable real-time identification and monitoring-based assessment of the bearing load state. The findings provide both a theoretical basis and a technical framework for evaluating the in-service performance of laminated elastomeric bridge bearings and for developing health-monitoring methodologies under varying thermal environments.

Compared with existing bearing monitoring approaches that mainly rely on embedded sensors, strain-based measurements, active sensing, or damage-identification signals, the present study focuses on an externally deployable laser displacement sensing method for load-state monitoring of laminated elastomeric bridge bearings under varying temperature conditions. The main contributions of this study are threefold. First, the temperature-dependent evolution of the compressive elastic modulus and vertical deformation response of a GBZY 300 × 63 laminated elastomeric bearing is systematically quantified over the temperature range from −20 °C to 30 °C. Second, the applicability of high-precision laser displacement sensors for monitoring the vertical compressive deformation of the bearing is evaluated through comparison with reference displacement transducers under both room-temperature and multi-temperature conditions. Third, a temperature-informed load inversion model is developed by incorporating both real-time displacement and temperature information, providing a practical framework for bearing load-state identification under thermal environments.

## 2. Materials and Methods

### 2.1. Material Preparation

**Figure 1:**
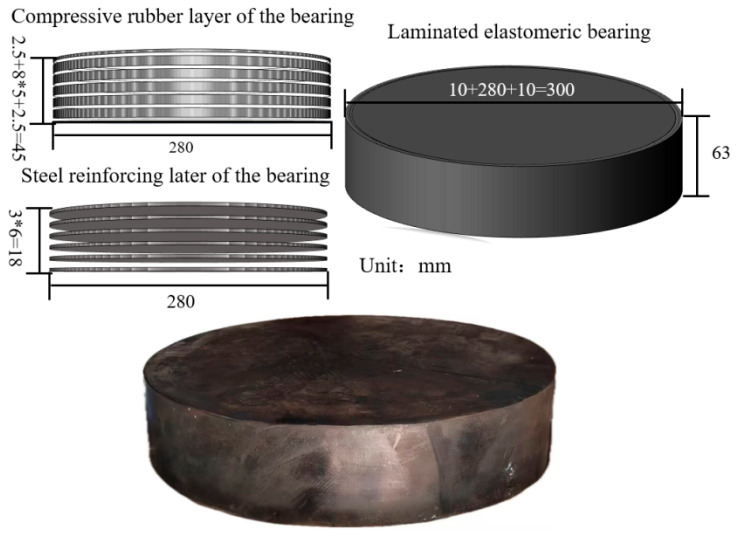
Structural diagram of the laminated elastomeric bearing.

A GBZY 300 × 63 laminated elastomeric bearing was selected as the test specimen in this study, with a design load-carrying capacity of 661 kN. The load-bearing region of the bearing consists of alternating layers of rubber and steel shims, forming a typical laminated configuration. In this specimen, six steel reinforcing plates are embedded, with a vertical spacing of 8 mm between adjacent plates. A schematic illustration of the bearing configuration is presented in Figure 1.

**Figure 2:**
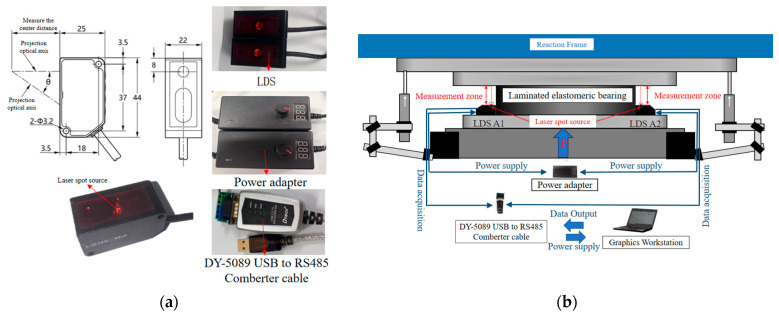
Measurement system of the LDS. (**a**) auxiliary components of the laser measurement system; (**b**) measurement configuration of the laser measurement system.

**Figure 3:**
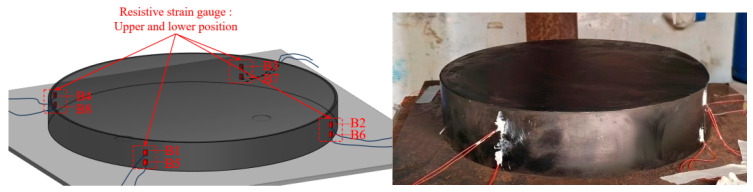
Layout of the electrical resistance strain gauges.

The experimental measurement system consisted primarily of laser displacement sensors (LDS), electrical resistance strain gauges, and displacement transducers. The LDS were PDL-200-485 units manufactured by Shenzhen Pineapple Automation Co., Ltd., (Shenzhen, China), with a measurement accuracy of 10 μm. Power was supplied through a DMD-2410 DC adapter. The sensors communicated with the workstation via half-duplex RS-485, and data transmission was implemented using a DY-5019 USB-to-RS485 converter (Guangzhou DTECH Electronics Technology Co., Ltd., Guangzhou, China). During testing, all data were acquired and recorded using the PD-Navigator_V4.3.3 data acquisition system; all associated accessories were provided by the manufacturer. The two LDS were denoted as A1 and A2. The strain gauges were labeled B1–B8 and bonded to the rubber layers located between the second and third, and fourth and fifth, internal steel shims of the laminated bearing. These gauges were used to monitor the strain response of the laterally bulged rubber regions during compressive loading. Specifically, B1–B4 were installed in the upper rubber layer, whereas B5–B8 were installed in the lower rubber layer. In each layer, the gauges were arranged symmetrically at the four corners. Because the external surface of the elastomeric bearing was relatively smooth, the bonding locations were mechanically roughened prior to installation in order to improve gauge adhesion and measurement stability. The Linear Variable Differential Transformer (LVDT) were labeled C1–C4. A schematic of the laser displacement measurement system is shown in Figure 2, and the layout of the strain gauges is presented in Figure 3.

### 2.2. Analysis of Test Conditions

Three categories of tests were conducted on the laminated elastomeric bearing in this study: sensor calibration tests at room temperature, compressive elastic modulus tests under different temperature conditions, and sensor temperature tests under different temperature conditions. The test temperature ranged from −20 to 30 °C, with six discrete temperature levels defined at 10 °C intervals. The room-temperature calibration tests were designed in accordance with JT/T 4-2019: Laminated Bearings for Highway Bridges [31].

The loading procedure was as follows. For all tests except the room-temperature calibration test, temperature conditioning was first performed. The specimen was placed in a constant-temperature chamber and adjusted to the target temperature using the thermal control system, while the specimen temperature was monitored continuously in real time. Once the difference between the actual specimen temperature and the preset value was controlled within 0.5 °C, the specimen was maintained at that temperature for at least 4 h to ensure a sufficiently stable internal temperature field. After thermal stabilization, preloading was applied using an electro-hydraulic servo loading system. Compressive stress was increased to 10 MPa at a loading rate of 0.03 MPa/s, held for 5 min, and then unloaded at the same rate back to the initial compressive stress, constituting one complete loading cycle. This procedure was repeated for three consecutive cycles in order to eliminate installation gaps within the test setup and at the contact interfaces between components.

During the formal testing stage, each test group underwent three calibration loading runs. In each formal loading sequence, every load level was maintained for 3 min, and once the measured response had stabilized, three consecutive readings were recorded and used as the test result for that specific condition. Based on the internal configuration and effective loaded area of the laminated elastomeric bearing, the applied average compressive stress was converted into an equivalent vertical load. Accordingly, an average compressive stress of 1 MPa corresponded to an equivalent vertical load of 70.6 kN, while 10 MPa corresponded to 706 kN. The test variables and parameters are summarized in Table 1.

**Figure 4:**
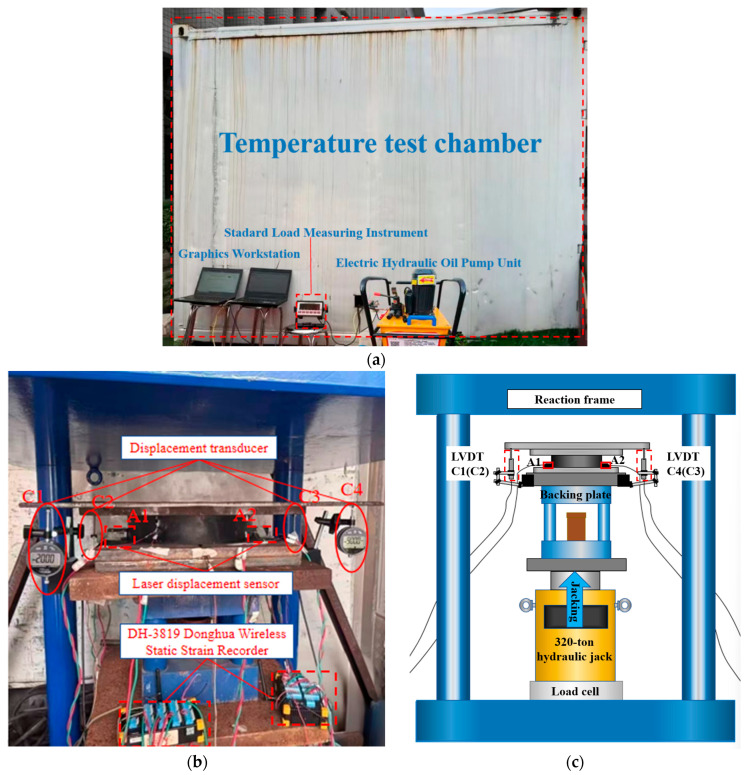
Test instruments and loading arrangement. (**a**) exterior view of the temperature test chamber; (**b**) instrument layout inside the chamber; (**c**) schematic of the loading setup.

Environmental temperature was simulated using a DK 92-5001 temperature chamber, with a controllable temperature range of −30 to 30 °C. Preliminary testing showed that when the chamber was set to −30 °C and maintained for 8 h, the actual stabilized temperature inside the chamber reached −27.2 °C. Considering both the operational limitations of the equipment and the requirement for stable temperature control, the temperature range adopted for the temperature-dependent tests in this study was ultimately defined as −20 to 30 °C. To accurately capture the actual temperature in the vicinity of the bearing during testing, a temperature-sensitive sensor was installed near the specimen inside the chamber, and the temperature data were recorded in real time using a VW-102 vibrating-wire readout unit. The measurement system consisted of a DH3819 Donghua wireless static strain acquisition instrument, a ZigBee wireless communication controller, displacement transducers, and a graphical workstation. The instrumentation layout and loading configuration are shown in Figure 4.

## 3. Results

### 3.1. Finite Element Analysis

The finite element analysis was used as a supporting mechanical analysis rather than as an independent thermo-mechanical simulation. Its main purpose was to examine the deformation pattern and stress/strain distribution of the laminated elastomeric bearing under vertical compression, thereby providing a mechanical basis for the subsequent sensor layout and displacement-based monitoring method.

To investigate the mechanical behavior of the laminated elastomeric bearing under a compressive stress of 10 MPa, a high-fidelity three-dimensional solid finite element model was developed using the APDL language in ANSYS (Mechanical APDL 2022 R1). The principal parameters of the finite element model are listed in Table 2.

**Figure 5:**
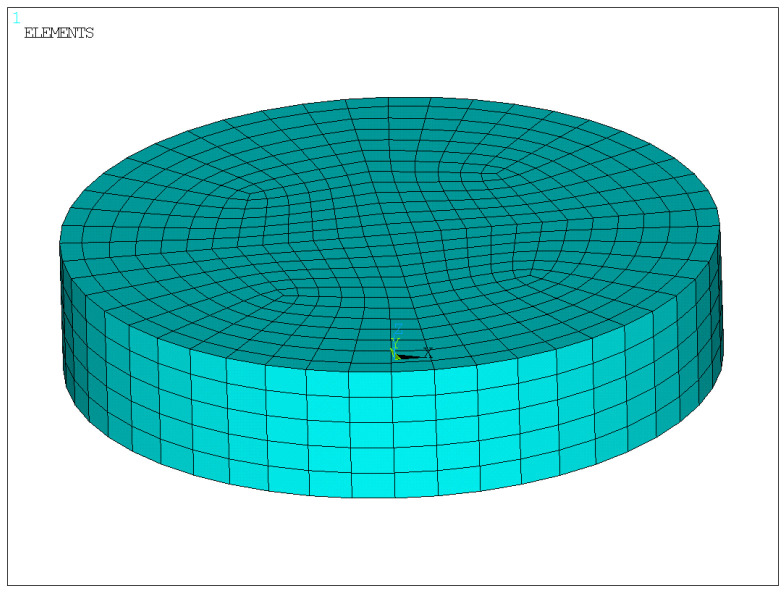
Finite element model.

**Figure 6:**
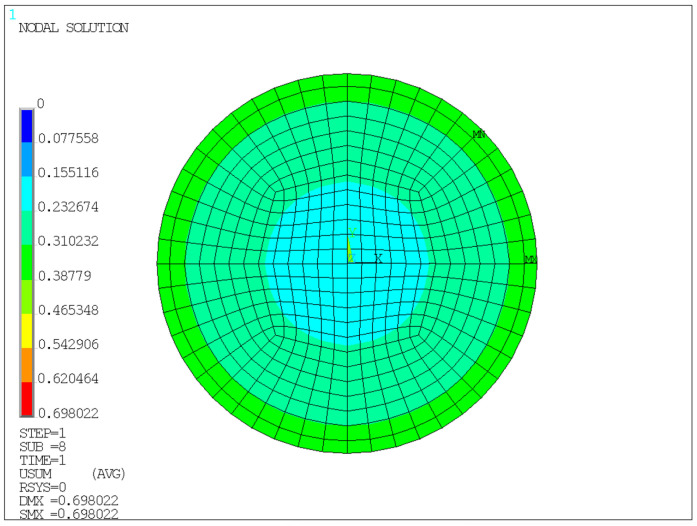
Total displacement contour of the front face under a compressive stress of 10 MPa.

**Figure 7:**
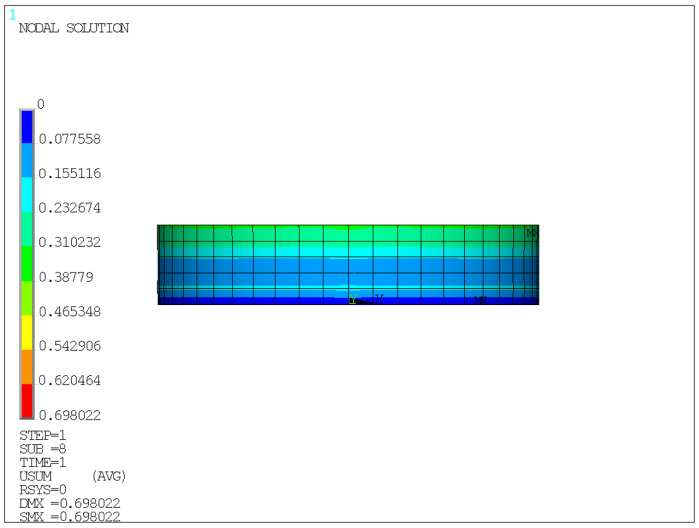
Total displacement contour of the side face under a compressive stress of 10 MPa.

**Figure 8:**
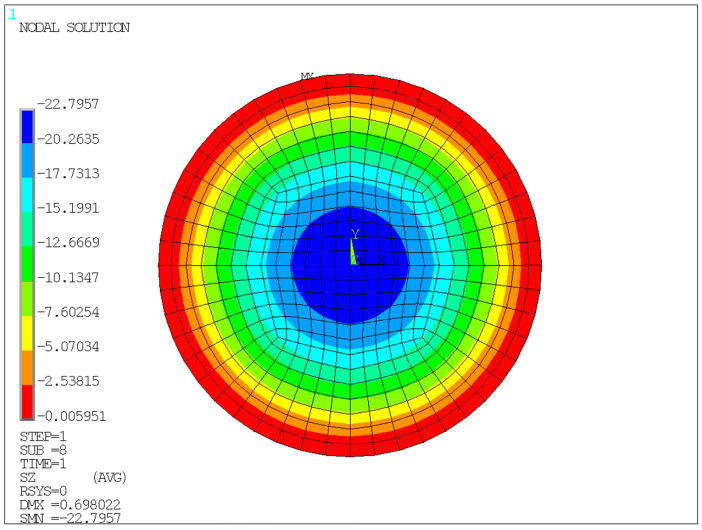
*Z*-axis stress contour of the front face under a compressive stress of 10 MPa.

**Figure 9:**
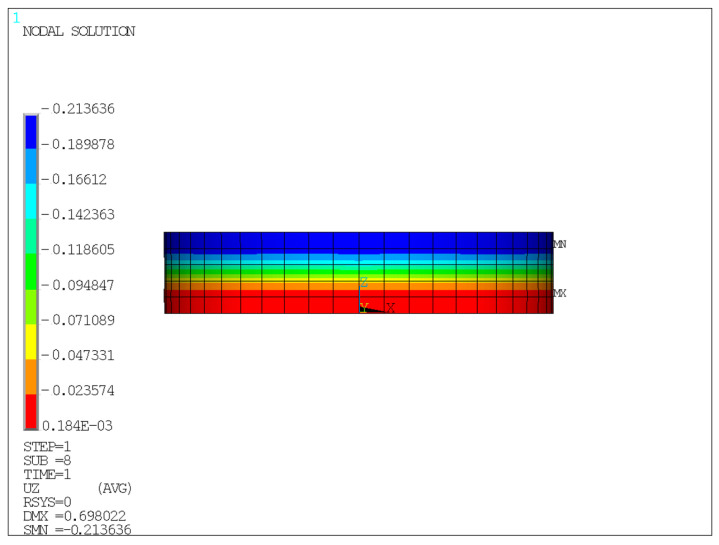
*Z*-axis strain contour of the side face under a compressive stress of 10 MPa.

The finite element model is shown in Figure 5, and the corresponding analysis results are presented in Figure 6, Figure 7, Figure 8 and Figure 9.

Unless otherwise specified, all contour plots were obtained under an average compressive stress of 10 MPa; displacement results are reported in mm, stress results are reported in MPa, and strain is dimensionless.

As shown in Figure 6, the total displacement on the front face of the bearing exhibits a distribution pattern characterized by larger values at the edges and smaller values in the central region, indicating that significant edge bulging deformation occurred in the rubber layers during vertical compression. This is mainly because the edge regions are subjected to weaker constraints, allowing deformation to be released more easily, whereas the central rubber is jointly restrained by the steel plates and the surrounding material, resulting in relatively smaller displacement. These results indicate that a pronounced edge effect exists in the bearing under compression. As shown in Figure 7, the total displacement on the side face of the bearing is unevenly distributed along the compression direction, with more obvious compressive displacement near the loading side. This indicates that load transfer has a certain directionality, and the internal compressive deformation is gradually transmitted and diffused along the loading path.

Figure 8 shows that the Z-direction stress in the rubber on the upper surface of the bearing is higher in the edge regions and lower in the central region, indicating that stress concentration is more likely to occur at the edges under vertical compression. This is related to the pronounced change in boundary conditions at the edges, as well as to the nearly incompressible nature of rubber, which tends to cause local stress accumulation when constrained by the steel plates.

Figure 9 indicates that the Z-direction strain on the side surface of the rubber is unevenly distributed along the height direction, being smaller in the upper region and larger in the lower region, and reaching its maximum near the fixed end. This suggests that boundary constraints have a significant influence on the internal strain distribution of the bearing, and that the region near the fixed end is the key area for local deformation and strain concentration.

According to the relevant provisions in Bridge Bearings [32] the compressive deformation of a laminated elastomeric bearing under an average compressive stress of 10 MPa should be controlled within 0.64–2.04% of its total thickness. For the bearing investigated in this study, this range corresponds to a vertical compressive deformation of 0.4032–1.2852 mm. The finite element results indicate that the vertical displacement of the bearing under 10 MPa compressive stress is 1.215 mm, which falls within the allowable range specified by the reference. This confirms that the compressive deformation response predicted by the developed model is reasonable and satisfies the required criterion.

Therefore, the finite element results support the experimental framework in two aspects. First, the calculated vertical compressive displacement under 10 MPa is consistent with the allowable deformation range and with the experimental measurements, indicating that the global compression response of the bearing is reasonable. Second, the simulated edge bulging and nonuniform stress/strain distribution explain why external displacement monitoring and local strain measurements can reflect the mechanical state of the bearing.

### 3.2. Room-Temperature Sensor Calibration Test

**Figure 10:**
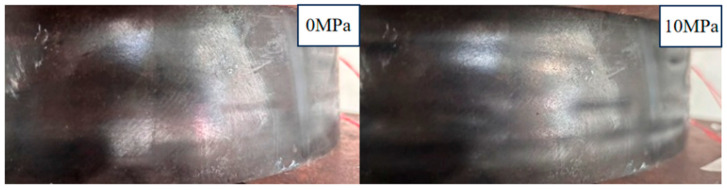
Experimental observations on the side face of the laminated elastomeric bearing.

**Figure 11:**
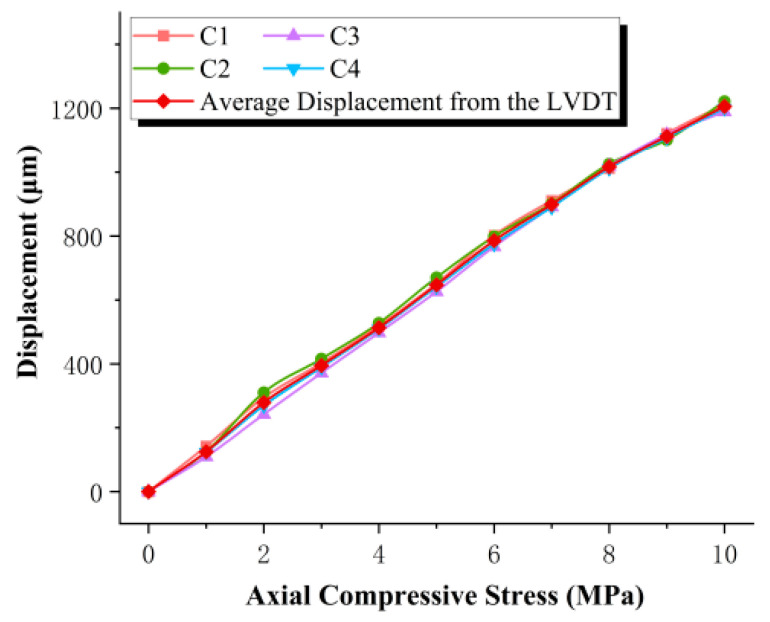
Average displacement measured by the LVDT.

**Figure 12:**
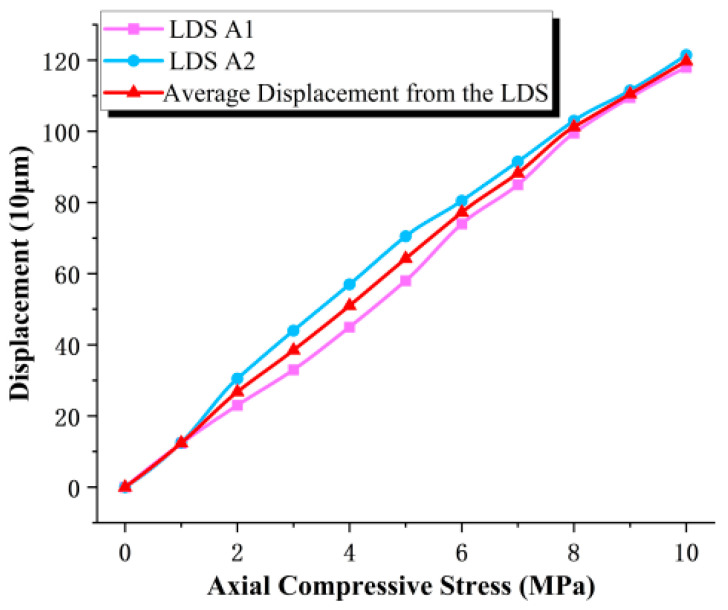
Average displacement measured by the LDS.

After installation of the strain gauges and LDS on the laminated elastomeric bearing, a room-temperature calibration test was conducted to evaluate the measurement performance of the LDS and electrical resistance strain gauges, using displacement transducers as reference instruments. During the test, an average compressive stress of 10 MPa was applied to the bearing. Under compression, the rubber layers in the load-bearing region exhibited pronounced lateral bulging deformation, as shown in Figure 10. The vertical calibration results obtained from the LVDT are presented in Figure 11, while those measured by the LDS are shown in Figure 12.

**Figure 13:**
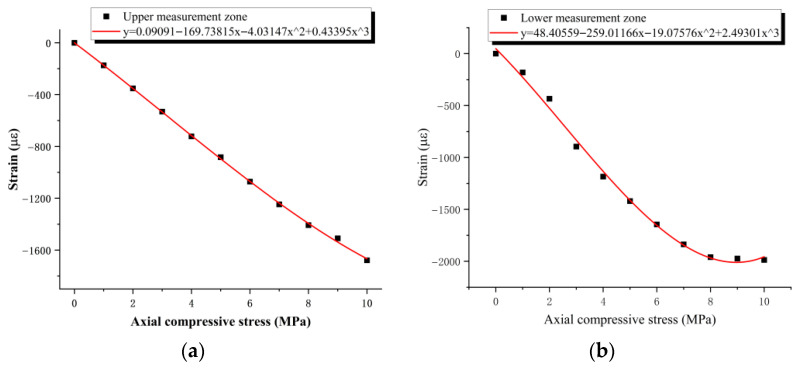
Average measured values of the electrical resistance strain gauges; (**a**) average strain in the upper measurement zone and fitted curve; (**b**) average strain in the lower measurement zone and fitted curve.

Under a vertical compressive stress of 10 MPa, pronounced lateral bulging was observed on the side surfaces of the laminated elastomeric bearing. This phenomenon arises because rubber is nearly incompressible; when subjected to vertical compression, the material accommodates the imposed deformation primarily through lateral expansion. However, the reinforcing steel shims between rubber layers constrain the transverse deformation of the internal rubber, causing the bulging deformation to concentrate mainly in the exposed outer rubber layers and at the bearing edges. Accordingly, this bulging behavior may be regarded as a typical mechanical response of laminated elastomeric bearings under compression and, within a certain loading range, as an indicator of normal working behavior. On this basis, electrical resistance strain gauges were used to monitor the strain response in the bulged regions of the upper and lower measurement zones, so as to compare the local strain behavior at different locations. The corresponding test results and fitted relationships are presented in Figure 13.

Under a compressive stress of 10 MPa, the experimentally measured vertical displacement of the elastomeric bearing was 1.184 mm, whereas the finite element prediction was 1.215 mm. The close agreement between these two values indicates that the finite element model is sufficiently accurate to capture the compressive deformation behavior of the bearing under vertical loading. In parallel, comparison between the LDS and LVDT measurements showed that, at 10 MPa, the average vertical displacement recorded by the LDS was 1.14 mm, differing from the average LVDT measurement by only 0.044 mm, corresponding to a relative error of 3.7%. These results demonstrate that the LDS provides adequate accuracy and practical applicability for monitoring the vertical compressive deformation of elastomeric bearings.

To characterize the strain behavior of the laminated elastomeric bearing at different elevations, electrical resistance strain gauges were installed at positions corresponding to one-third and two-thirds of the bearing height. Based on the experimental data, the response curves were fitted using the least-squares method, yielding an excellent fit with a coefficient of determination of R^2^ = 0.9955. The resulting fitted expressions are given in Equations (1) and (2).

Y = 0.09091 − 169.73815x − 4.03147x^2^ + 0.43395x^3^ (1)

Y = 48.40559 − 259.01166x − 19.07576x^2^ + 2.4930x^3^ (2)

where x denotes the axial compressive stress and Y represents the measured strain.

As shown in Figure 12, under the same vertical load, the strain response in the lower measurement zone was consistently greater than that in the upper measurement zone, indicating a pronounced spatial variation in local deformation along the bearing height, with more significant deformation concentrated in the lower region. Compared with the finite element analysis, although the compressive deformation of the bearing under actual working conditions is manifested as compression on both sides, it still exhibits a pronounced loading-path effect, with more significant local deformation on the loading side. It should be noted, however, that under cyclic loading, strain gauges bonded to the rubber surface are prone to debonding or damage, which can adversely affect the stability and repeatability of the measurements. For this reason, electrical resistance strain gauges were not used in the subsequent tests to monitor the lateral compressive deformation of the bearing.

For elastomeric bearings under long-term sustained loading, bulging deformation and material creep are intrinsically coupled, leading to the progressive accumulation of both vertical compressive deformation and lateral bulging, as well as to increased secondary shear strain and elevated stress levels in the internal steel shims. Once these time-dependent effects develop beyond a certain level, the load-transfer performance of the bearing may be adversely affected, potentially inducing service-related problems such as deck misalignment between adjacent spans, redistribution of internal forces in the girder, and edge cracking or delamination of the bearing. To address this issue, the present study employs the LDS to monitor the compressive displacement at the two symmetric ends of the bearing and adopts 5% of the bearing thickness as the threshold for compression-failure assessment, thereby evaluating whether the bearing has entered a crushing state.

### 3.3. Temperature-Dependent Compressive Elastic Modulus Test

**Figure 14:**
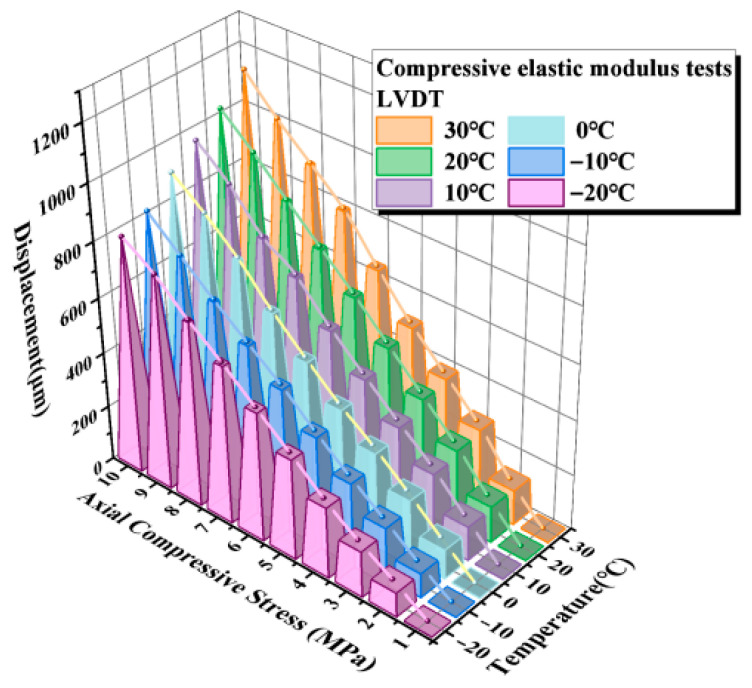
Compressive elastic modulus determined from LVDT measurements.

**Figure 15:**
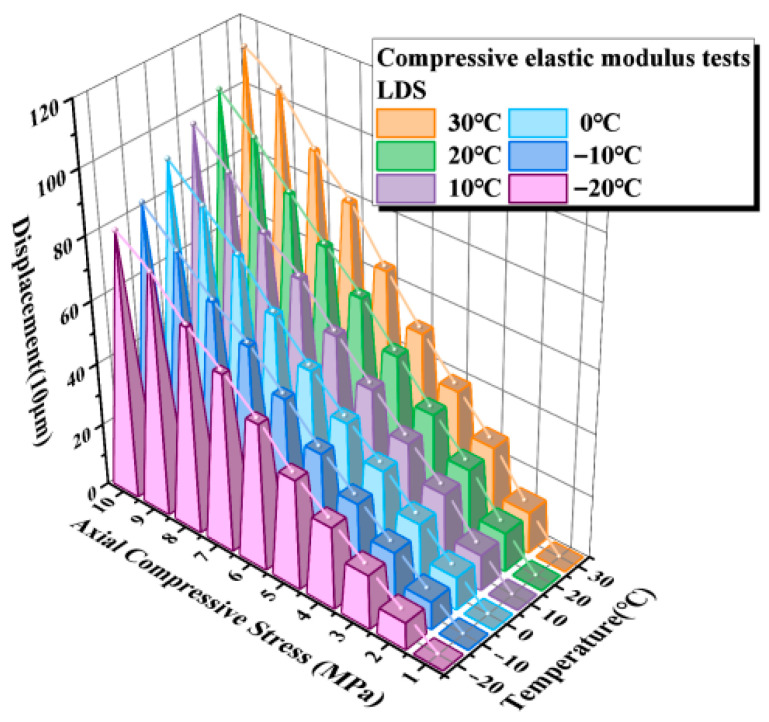
Compressive elastic modulus determined from LDS measurements.

The compressive elastic modulus tests in this section were conducted in accordance with the relevant provisions of JT/T 4-2019, Laminated Elastomeric Bearings for Highway Bridges [31]. The loading protocol started from an initial compressive stress of 1 MPa and was then increased incrementally to 10 MPa. The results obtained from the displacement transducers are presented in Figure 14, while those measured by the LDS are shown in Figure 15.

As shown in Figure 14 and Figure 15, the load–displacement responses of the elastomeric bearing under vertical compression exhibit broadly consistent trends across the six discrete temperature conditions, indicating good repeatability and stability of the experimental results. Under the maximum compressive stress of 10 MPa, the maximum difference between the average displacements measured by the LVDT and the LDS was 3.54%, demonstrating that the LDS maintains a high level of displacement-monitoring accuracy even under varying temperature conditions. Further analysis reveals that the vertical compressive displacement of the bearing decreases progressively as the ambient temperature is reduced, indicating that low temperature suppresses the compressive deformability of the bearing. This behavior can be primarily attributed to the temperature-induced increase in rubber stiffness and compressive elastic modulus, which gives rise to a pronounced low-temperature stiffening effect in the bearing.

In summary, ambient temperature has a pronounced effect on the vertical compressive response of elastomeric bearings. Under low-temperature conditions, the compressive stiffness of the bearing increases, while its vertical deformability is correspondingly restrained, leading to a marked reduction in compressive displacement. These results demonstrate that the vertical mechanical behavior of elastomeric bearings exhibits strong temperature sensitivity.

### 3.4. Sensor Temperature Test

**Figure 16:**
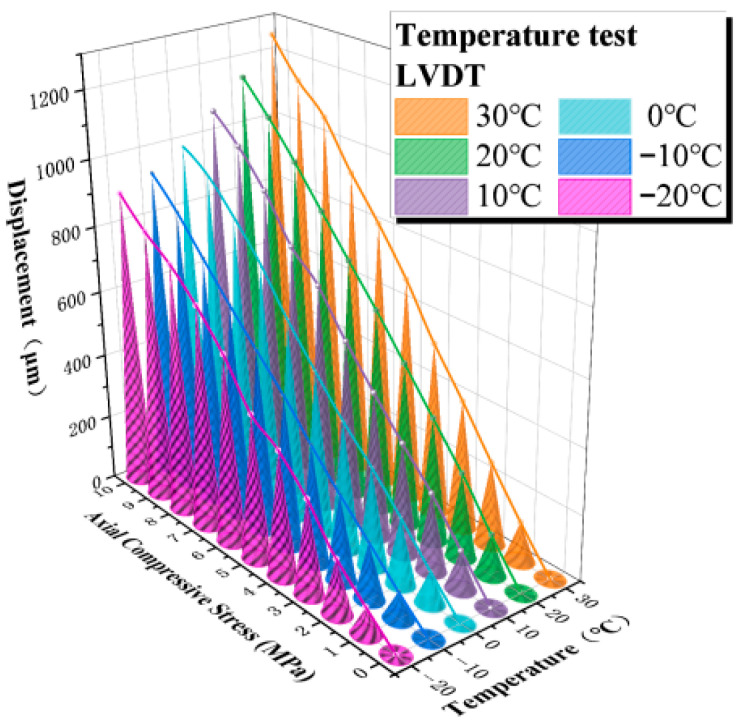
Average test values measured by the LVDT.

**Figure 17:**
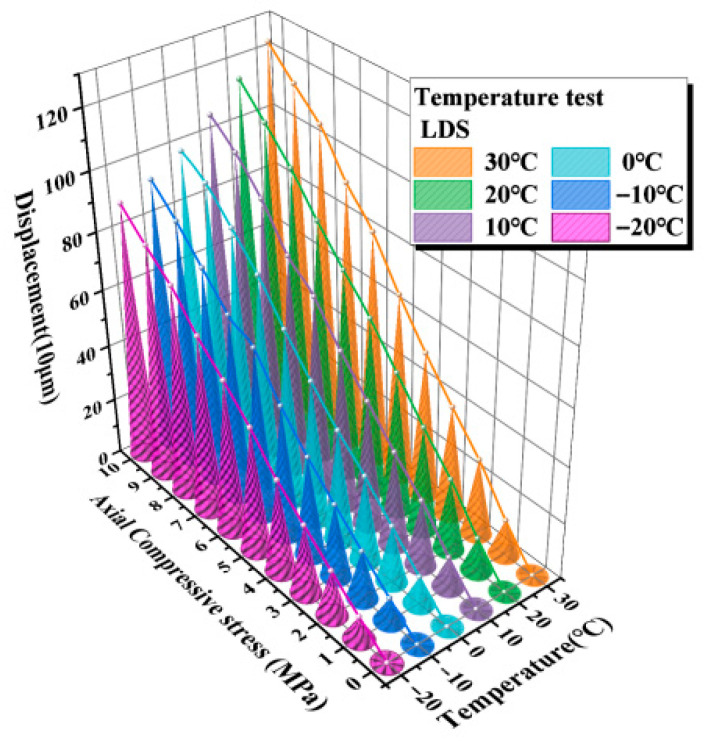
Average test values measured by the LDS.

The results of the compressive elastic modulus tests indicate that the compressive deformation response of the elastomeric bearing is highly sensitive to ambient temperature. On this basis, to further verify the feasibility and applicability of the LDS for deformation monitoring of elastomeric bearings under different thermal conditions, temperature tests were conducted using simultaneous measurements from the displacement transducers and the LDS. The corresponding sensor results for each temperature condition were obtained accordingly. The measurements from the displacement transducers are shown in Figure 16, and those from the LDS are presented in Figure 17.

As shown in Figure 16 and Figure 17, the vertical displacement response of the elastomeric bearing exhibits a clear temperature-dependent stratification across the different temperature levels. With increasing compressive stress, the vertical compressive displacement increases continuously under all thermal conditions; however, the displacement–stress curves differ markedly from one temperature level to another, indicating that ambient temperature directly affects the deformation response of the bearing during loading. The displacement trends measured by the displacement transducers and the LDS are generally consistent at all temperature levels, demonstrating that the LDS can effectively track the vertical deformation evolution of the elastomeric bearing under varying thermal conditions.

Under the same compressive stress level, the vertical displacement of the bearing under low-temperature conditions is consistently smaller than that under higher-temperature conditions, indicating that a reduction in temperature markedly suppresses the compressive deformation of the bearing. Taking the 10 MPa loading condition as an example, the displacement transducers recorded vertical displacements of 1.258 mm at 30 °C and 0.903 mm at −20 °C, corresponding to a difference of 0.355 mm and a reduction of 28.2%. The corresponding displacements measured by the LDS were 1.24 mm and 0.90 mm, respectively, giving a difference of 0.338 mm and a reduction of 27.42%. The two measurement methods show good agreement in both trend and magnitude, further confirming that low-temperature environments significantly reduce the compressive deformability of elastomeric bearings while enhancing their overall resistance to deformation.

## 4. Discussion

### 4.1. Analysis of the Temperature Effect on the Compressive Elastic Modulus

Because the Poisson’s ratio of rubber is close to 0.5, the material can be regarded as nearly incompressible under compression. Therefore, within a certain stress range, it is commonly approximated as a linear elastic material for mechanical analysis. According to JT/T 4-2019, Laminated Bearings for Highway Bridges [31], the empirical expression for the compressive elastic modulus E of a circular laminated elastomeric bearing is given in Equation (3), whereas the expression used in this study to calculate the compressive elastic modulus $E_{1}$ from the experimental results is presented in Equation (5).

(3) $$E=5.4GS^{2}$$

(4) $$S=\frac{d_{0}}{4t_{1}}$$

where E is the empirical value of the compressive elastic modulus; G is the shear modulus, which is typically taken as 1.0 MPa at room temperature; S is the shape factor; $d_{0}$ is the effective compressed diameter; and $t_{1}$ is the thickness of a single internal rubber layer.

(5) $$E_{1}=\frac{\sigma_{10}-\sigma_{4}}{\varepsilon_{10}-\varepsilon_{4}}$$

where $\sigma_{10}$ is the compressive stress corresponding to the 10 MPa loading level, $\sigma_{4}$ is the compressive stress corresponding to the 4 MPa loading level, $\varepsilon_{10}$ is the accumulated compressive strain at the 10 MPa loading level, and $\varepsilon_{4}$ is the accumulated compressive strain at the 4 MPa loading level.

Based on the geometric parameters of the elastomeric bearing and the experimentally measured values, the compressive elastic modulus at each temperature level was calculated using Equations (3) and (5). The corresponding results are summarized in Table 3.

According to the empirical formula, the compressive elastic modulus of the elastomeric bearing was calculated to be 413.44 MPa, whereas the experimentally determined value at 30 °C was 464 MPa, representing an increase of approximately 12.2% over the empirical estimate. This discrepancy may be attributed to several factors. First, the actual shear modulus of the rubber material may be higher than the nominal value commonly adopted in the specification. Second, deviations in the thickness of individual rubber layers may have arisen during manufacturing. Third, differences may exist between the effective compressed dimensions of the bearing used in the experiment and those assumed in the theoretical model. Since the observed deviation remains within the 20% allowable tolerance specified for the measured compressive elastic modulus, the result is considered reasonable, indicating that the actual vertical compressive stiffness of the bearing is slightly higher than the theoretical prediction.

Compared with the value at 30 °C, the compressive elastic modulus increases by 9.48%, 13.58%, 18.97%, 26.94%, and 32.11% at 20 °C, 10 °C, 0 °C, −10 °C, and −20 °C, respectively. Over the temperature range from 30 °C to −20 °C, the compressive elastic modulus increases by an average of 6.422% for every 10 °C decrease in temperature, further demonstrating that low-temperature conditions significantly enhance the resistance of elastomeric bearings to compressive deformation.

These results confirm the temperature sensitivity and low-temperature stiffening behavior of the laminated elastomeric bearing. Given that rubber serves as the primary load-bearing medium in the bearing system and that its mechanical behavior is highly temperature-sensitive, temperature effects must be explicitly accounted for in bridge health monitoring and bearing performance assessment in order to improve the reliability of monitoring interpretation and condition identification.

For bearings with different dimensions or rubber formulations, the temperature-dependent calibration relationship should not be directly transferred without additional verification. The absolute value of the compressive elastic modulus is affected by the bearing geometry, shape factor, rubber compound, manufacturing process, and aging condition. However, the calibration strategy proposed in this study can be transferred in a methodological sense. Specifically, for a new bearing type, multi-temperature compression tests can be conducted to identify the temperature-dependent modulus variation, and the corresponding temperature-dependent coefficients can then be recalibrated for the load inversion model. Therefore, the present model coefficients are specific to the tested GBZY 300 × 63 bearing, whereas the overall calibration and inversion framework can be extended to other bearing types after appropriate recalibration.

### 4.2. Temperature-Informed Calculation Method for Compressive Stress and Load-Carrying Capacity of Elastomeric Bearings

**Figure 18:**
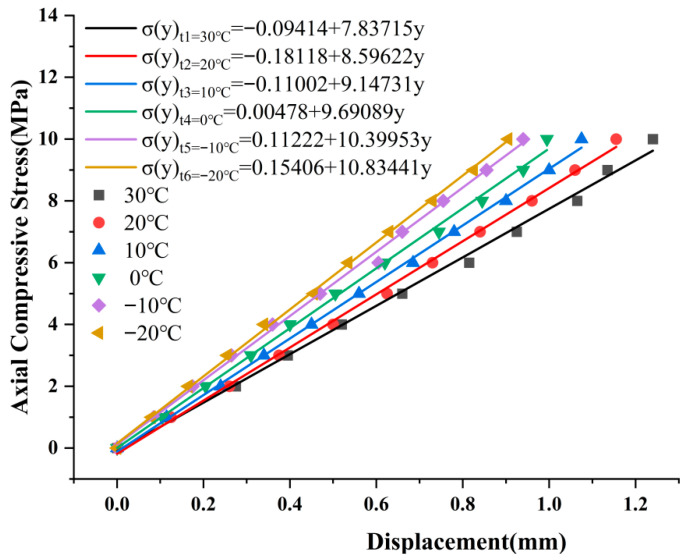
Temperature test data from the LDS and the fitted equation.

The temperature calibration results indicate that the LDS exhibits good stability and reliability in monitoring the vertical compressive deformation of elastomeric bearings and is therefore capable of meeting the displacement-measurement requirements under large-deformation conditions. On this basis, and to further evaluate its applicability across different temperature levels, a real-time inversion model for the LDS was established using the experimental data and was subsequently employed to identify the load-carrying capacity of the elastomeric bearing through inverse analysis. The corresponding temperature test results and fitted relationships are presented in Figure 18. Because no significant temperature-induced interference was observed in the sensor ranging results within the temperature range considered in this study, no additional temperature compensation was applied to the measured data in the subsequent analysis.

Based on the fitting results shown in Figure 18, the relationship between the average compressive stress and the measured displacement of the sensor can be expressed as Equation (6):

(6) $${\sigma (y)}_{t_{k}}=\sum_{i=0}^{n}a_{\left(i,t_{k}\right)}{y_{L}}^{i}$$

Based on the temperature test data of the sensor, the displacement–average compressive stress relationship was fitted by linear regression using the least-squares method. When the coefficient of determination of the fitted equation satisfied R^2^ > 0.99, setting i = 1 was sufficient to meet the required fitting accuracy. Accordingly, Equation (6) can be further expanded as follows:

(7) $$\left\{\begin{array}{c} {\sigma (y)}_{t_{1}}=a_{\left(0,t_{1}\right)}+a_{\left(1,t_{1}\right)}y_{L}\\ \begin{array}{c} \begin{array}{ccc} \vdots & \vdots & \vdots \end{array}\\ {\sigma (y)}_{t_{6}}=a_{\left(0,t_{6}\right)}+a_{\left(1,t_{6}\right)}y_{L} \end{array} \end{array}\right.$$

As shown in Figure 18, the coefficient of the first-order term, denoted as $a_{(i,t_{k})}$, in the fitted equation decreases progressively as the temperature decreases. To account for the influence of temperature on the equation coefficients, the relationship between $a_{(i,t_{k})}$ and the discrete temperature levels was further fitted using the least-squares method, and a fitted expression describing the dependence of the temperature–compressive stress correlation coefficient $a_{(i,t_{k})}$ on temperature was established. The corresponding formulation is given in Equation (8).

(8) $$a_{\left(i,t_{k}\right)}=\sum_{j=0}^{m}b_{\left(i,j\right)}T^{f}$$

Using the least-squares method, regression fitting was performed for the coefficient $a_{(i,t_{k})}$. The results show that, for $a_{(0,t_{k})}$, when the fitting order is taken as f = 3, the coefficient of determination satisfies R^2^ > 0.99. Likewise, for $a_{(1,t_{k})}$, when the fitting order is taken as f = 2, the fitted relationship also satisfies R^2^ > 0.99. Accordingly, the constant term and the first-order term in Equation (7) can be further expressed as Equation (9).

(9) $$\begin{array}{c} a_{\left(0,t_{k}\right)}=b_{\left(0,0\right)}+b_{\left(0,1\right)}T+b_{\left(0,2\right)}T^{2}+b_{\left(0,3\right)}T^{3}\\ a_{\left(1,t_{k}\right)}=b_{\left(1,0\right)}+b_{\left(1,1\right)}T+b_{\left(1,2\right)}T^{2} \end{array}$$

where ${\sigma (y)}_{t_{k}}$ denotes the calculated load; k represents the temperature level, taking values of 1, 2, 3, 4, 5, and 6, which correspond to 30 °C, 20 °C, 10 °C, 0 °C, −10 °C, and −20 °C, respectively; $a_{(i,t_{k})}$ is the temperature–compressive stress correlation coefficient; $b_{\left(i,j\right)}$ is the temperature–strain correlation coefficient; $y_{L}$ denotes the average measured displacement; and T is the actual measured temperature.

By combining Equations (7) and (9), the stress inversion formula for the LDS can be obtained, as expressed in Equation (10):

(10) $$\begin{array}{c} \sigma (y)=0.00488-0.013T-0.00004138T^{2}+0.000012025T^{3}\\ +(9.7673-0.05832T-0.000162365T^{2})y \end{array}$$

In Equation (10), the displacement output y of the LDS is input in mm, whereas the temperature variable T is input in °C.

The selected fitting forms were determined according to both fitting accuracy and model simplicity. For each temperature level, the relationship between the measured vertical displacement and the average compressive stress was approximately monotonic and quasi-linear within the investigated loading range; therefore, a first-order displacement–stress relationship was first adopted. For the temperature-dependent coefficients, polynomial fitting was performed using the least-squares method, and the lowest polynomial order satisfying R^2^ > 0.99 was selected. This strategy was adopted to avoid unnecessary overfitting while maintaining sufficient accuracy for engineering monitoring applications. Accordingly, the proposed inversion formula is empirical and should be used only within the calibrated temperature range of −20 °C to 30 °C and the stress range of 0–10 MPa.

To verify the feasibility of the proposed stress inversion model, model validation was carried out using the vertical calibration test data obtained at room temperature (25.2 °C). The inverted stress values were then converted into equivalent loads according to the relationship that the bearing compressive stress of 1 MPa corresponds to 70.6 kN. Subsequently, the experimental data were substituted into Equation (10) to calculate the vertical compressive stress of the bearing and the corresponding relative error. The calculated results are presented in Table 4.

As shown in Table 4, the maximum relative error of the LDS-based inversion results within the 0–10 MPa compressive stress range is 4.83%, which is below 5%, indicating that the proposed model provides good accuracy for monitoring the vertical compressive stress of the bearing. Under room-temperature conditions, the relative errors of the inverted results for all loading cases remained within an acceptable range, further confirming the reliability of the proposed inversion model. It should be noted that the largest relative error occurred near the zero-load condition. This is mainly because the denominator in the relative-error calculation is very small at low stress levels; therefore, even a small absolute deviation may result in an apparently large relative error. In addition, an average residual displacement of 0.0250 mm was observed at 0 MPa after three loading cycles, indicating incomplete recovery of the specimen after unloading due to the viscoelastic behavior of rubber. Therefore, the relative error near zero load should be interpreted with caution. In this study, a rest period of no less than 10 min was introduced after each loading cycle to reduce the influence of accumulated residual deformation on subsequent measurements. For practical load-monitoring applications, the model is expected to show better stability within the main working load range of the bearing.

The uncertainty of the proposed inversion method mainly originates from sensor measurement error, temperature-control error, residual deformation of the specimen, and regression fitting error. In the present tests, the LDS had a measurement accuracy of 10 μm, and the specimen temperature was controlled within ±0.5 °C of the target value after thermal stabilization. At each load level, three consecutive readings were recorded after the response stabilized to reduce random measurement fluctuations. The maximum average displacement difference between the LDS and the LVDT under multi-temperature conditions was 3.54%, and the maximum relative error of the inverted stress in the independent room-temperature validation test was 4.83%. These results provide a preliminary assessment of the overall uncertainty of the monitoring framework. A more comprehensive statistical uncertainty analysis based on multiple specimens and independent validation datasets will be conducted in future work.

## 5. Conclusions

This study investigated the vertical compressive mechanical response and the corresponding load-monitoring method of a GBZY 300 × 63 laminated elastomeric bearing over a temperature range of −20 to 30 °C, based on high-precision laser displacement sensing technology. The main conclusions are as follows:

(1) The high-precision LDS can accurately monitor the vertical compressive displacement of laminated elastomeric bearings. Under both room-temperature and multi-temperature conditions, the measurements obtained from the LDS showed good agreement with those from the LVDT, with a maximum average displacement error of 3.54%, demonstrating its high measurement accuracy, stability, and engineering applicability.
(2) Ambient temperature has a pronounced effect on the vertical compressive mechanical behavior of laminated elastomeric bearings. As the temperature decreases, the vertical compressive displacement of the bearing gradually decreases, while the compressive elastic modulus increases continuously, indicating a clear low-temperature stiffening effect. At −20 °C, the vertical displacement was reduced by approximately 28.2% relative to that at 30 °C, whereas the compressive elastic modulus increased by 32.11%.
(3) The load inversion model established on the basis of real-time temperature and displacement response exhibits high identification accuracy. Within the investigated temperature range, the model achieved a maximum relative error of 4.83%, demonstrating its capability to effectively identify the load-bearing state of the bearing. The results indicate that explicit incorporation of temperature effects into the monitoring model is essential for improving the accuracy of load-state identification in bridge bearings.

Overall, temperature effects significantly influence both the mechanical response of laminated elastomeric bearings and the interpretation of monitoring results. The methodology proposed in this study can provide a useful reference for the performance evaluation and structural health monitoring of bridge elastomeric bearings operating under complex environmental conditions.

It should be noted that the present experimental program was conducted on a representative GBZY 300 × 63 laminated elastomeric bearing. Although repeated loading runs, multiple load levels, and six temperature conditions were adopted to improve the reliability of the test results, the influence of specimen-to-specimen variability, bearing size, rubber compound, manufacturing batch, aging state, and long-term field exposure was not fully covered in this study. Therefore, the proposed temperature-informed load inversion method should be interpreted as a validated framework for the investigated bearing configuration and temperature range, rather than as a universal model for all laminated elastomeric bearings. Further studies involving multiple bearing specimens and field monitoring data are needed to evaluate the broader applicability and robustness of the method.

## Figures and Tables

**Table 1:** Test types and experimental variables.

Test Type	Temperature Level (°C)	Loading Path: Stress (MPa)/Load (kN)	Data Acquisition at Each Load Level
Test 1 (11 Loading Levels)	25.2 (Room Temperature)	Stress: 0→1→…→10 Load: 0→70.6→…→706	Three readings recorded after 3 min of load holding (1 s interval)
Test 2 (10 Loading Levels)	−20, −10, 0, 10, 20, 30	Stress: 1→2→…→10 Load:70.6→141.2→…→706	
Test 3 (11 Loading Levels)	−20, −10, 0, 10, 20, 30	Stress: 0→1→…→10 Load: 0→70.6→…→706	

Note: Test 1 denotes the room-temperature sensor calibration test; Test 2 denotes the temperature-dependent compressive elastic modulus test; and Test 3 denotes the sensor temperature test.

**Table 2:** Model parameters of the laminated elastomeric bearing.

Structural Component Type	Material Type	Element Type	Elastic Modulus (MPa)	Poisson’s Ratio
Rubber	natural rubber	Solid185	3.375 × 10^3^	0.4998
Reinforcing steel shim	Q235C	Solid185	2.1 × 10^5^	0.2900

**Table 3:** Compressive elastic modulus at different temperature levels.

Temperature (°C)	Calculated Bearing Compressive Elastic Modulus (MPa)
30	464
20	508
10	527
0	552
−10	589
−20	613

**Table 4:** Relative error of the load calculated from the LDS test at room temperature (25.2 °C).

Measurement Source	Applied Stress (MPa)	Average Displacement (mm)	Predicted Stress (MPa)	Predicted Load (kN)	Relative Error in Stress Prediction (%)
Average Value Measured by the LDS	0	0.0250	0.0483	3.41	4.83
	1	0.1400	0.9907	69.94	0.93
	2	0.2750	2.0355	143.75	1.78
	3	0.3900	2.9983	211.69	0.06
	4	0.5300	4.0226	284.00	0.57
	5	0.6525	5.1084	360.65	2.17
	6	0.7725	6.1737	435.86	2.90
	7	0.8825	7.0751	499.45	1.07
	8	1.0125	8.1404	574.71	1.76
	9	1.0975	8.8369	623.89	1.81
	10	1.1975	9.6564	681.93	3.44

## Data Availability

Data is unavailable due to privacy.

## References

[1] Azimi M., Eslamlou A.D., Pekcan G. (2020). Data-Driven Structural Health Monitoring and Damage Detection through Deep Learning: State-of-the-Art Review. Sensors.

[2] Sun L.-M., Shang Z.-Q., Xia Y., Bhowmick S., Nagarajaiah S. (2020). Review of Bridge Structural Health Monitoring Aided by Big Data and Artificial Intelligence: From Condition Assessment to Damage Detection. J. Struct. Eng..

[3] Yu Z.-R., Shan D.-S., Sun R.-H. (2025). Population-Based Structural Health Monitoring of Bridges: Review and Challenges. J. Traffic Transp. Eng..

[4] Wang Q., Pan Y.-J., Deng L., Ju X.-C. (2025). Research and Application of Intelligent Monitoring Technology for Bearing Displacement in Long-Span Railway Bridges. Railw. Eng..

[5] Wang S., Wu Q., Li Y., Zi D., Wei L., Liang Y. (2025). Experimental Investigation on Mechanical and Aging Properties of Novel High Durability NR/EPDM Blended Rubber Bearings. Constr. Build. Mater..

[6] Xiang N., Li J. (2017). Experimental and Numerical Study on Seismic Sliding Mechanism of Laminated-Rubber Bearings. Eng. Struct..

[7] Liang Y., Xiong F. (2020). Measurement-Based Bearing Capacity Evaluation for Small and Medium Span Bridges. Measurement.

[8] Zheng Y., Yue C. (2020). Shaking Table Test Study on the Functionality of Rubber Isolation Bearing Used in Underground Structure Subjected to Earthquakes. Tunn. Undergr. Space Technol..

[9] Zhang R.-J., Li A.-Q. (2020). Experimental Study on Temperature Dependence of Mechanical Properties of Scaled High-Performance Rubber Bearings. Compos. Part B Eng..

[10] Yakut A., Yura J.A. (2002). Parameters Influencing Performance of Elastomeric Bearings at Low Temperatures. J. Struct. Eng..

[11] You S.-Q., Liu B., Lou Y.-L. (2005). Low-Temperature Effect on Deformation Behavior of Laminated Rubber Isolators. J. Northeast. Univ..

[12] Chen Z., Huang H.-Z., Deng Z., Wu J. (2025). Shrinkage Mamba Relation Network with Out-of-Distribution Data Augmentation for Rotating Machinery Fault Detection and Localization under Zero-Faulty Data. Mech. Syst. Signal Process..

[13] Chen Z., Wu J., Deng Z., Huang H.-Z. (2026). Learning Category-Invariant Disentangled Features for Domain Generalization in Machine Fault Diagnosis. IEEE/ASME Trans. Mechatron..

[14] Casciati F., Faravelli L. (2012). Experimental Investigation on the Aging of the Base Isolator Elastomeric Component. Acta Mech..

[15] Zeng Y., He Z., Pan P. (2023). A Deep Learning Method to Monitor Axial Pressure and Shear Deformation of Rubber Bearings under Coupled Compression and Shear Loading. Earthq. Eng. Struct. Dyn..

[16] Wu H.-Q., Chen J.-X., Li W.-Z., Hu Q., Liu X.-H. (2022). Design and Performance Study of an Intelligent Pot Rubber Bearing. J. Guangxi Univ. Sci. Technol..

[17] Tian Y.-S., Wu H.-Q., Hu Q., Li W.-Z., Liu X.-H. (2021). Research on Intelligentization of Plate Rubber Bearing. J. Guangxi Univ. Sci. Technol..

[18] Deng N., He M., Gu N., Liang H. (2024). Design and Performance Research of a New Type of Spherical Force-Measuring Bearing of Bridges Based on Button Type Microsensor. KSCE J. Civ. Eng..

[19] Imbimbo M., De Luca A.F.E. (1998). Stress Analysis of Rubber Bearings under Axial Loads. Comput. Struct..

[20] Ai D., Yang K. (2025). Axial Pressure and Damage Identification of Plate Rubber Bearings Using Transfer Learning of Electromechanical Admittance Signals from Different PZT Transducers. Eng. Struct..

[21] Zeng Y., Wang H., Deng K., Pan P. (2022). Detection of Rupture inside Rubber Bearings Using Active Sensing Method. Eng. Struct..

[22] Zeng Y., Pan P., Cao Y., He Z. (2021). Shear Deformation Detection in Smart Rubber Bearing (SRB) Using Active Sensing Method. Eng. Struct..

[23] Gauron O., Saidou A., Busson A., Siqueira G.H., Paultre P. (2018). Experimental Determination of the Lateral Stability and Shear Failure Limit States of Bridge Rubber Bearings. Eng. Struct..

[24] Pang H., Jiang T., Dai J., Yang Y. (2025). Study the Effects of Ambient Temperature on Full-Scale Rubber Bearings. Structures.

[25] Liu W.-G., Qin H.-T., He W.-F., Feng D.-M., Wang H.-Q. (2012). Mechanical Properties of LRB in Low Temperature State and Its Influence on Earthquake Response of High Buildings. J. Vib. Shock.

[26] Yasar C., Karuk V., Kaplan O., Cavdar E., Ozdemir G. (2023). Amplification in Mechanical Properties of a Lead Rubber Bearing for Various Exposure Times to Low Temperature. Buildings.

[27] Fuller K.N.G., Gough J., Thomas A.G. (2010). The Effect of Low Temperature Crystallization on the Mechanical Behavior of Rubber. J. Polym. Sci. B Polym. Phys..

[28] Ma Z., Guan Z., Li X. (2025). Design Method of Seismic Isolation Bridges with Lead-Core Rubber Bearings Considering Temperature Sensitivity. J. Vib. Shock.

[29] Liu B., Wang Y., Zhu Z., Theodorakis P.E., Song J., Bennacer R. (2023). A Lower Temperature Difference of the Elastocaloric Effect by Natural Rubber. Int. J. Refrig..

[30] Bennacer R., Liu B., Yang M., Chen A. (2022). Refrigeration Performance and the Elastocaloric Effect in Natural and Synthetic Rubbers. Appl. Therm. Eng..

[31] (2019). Laminated Bearing for Highway Bridge.

[32] Zhuang J.-S. (2015). Bridge Bearings.

